# Iris melanoma associated with unilateral phthisis bulbi in a 13-year-old domestic shorthair female cat

**DOI:** 10.1080/01652176.2019.1655604

**Published:** 2019-08-10

**Authors:** Barbara Lamagna, Valeria Uccello, Francesco Prisco, Valeria Russo, Francesco Lamagna, Luigi Navas, Giuseppina Mennonna, Carla Murino, Leonardo Meomartino

**Affiliations:** a Surgery Unit of the Department of Veterinary Medicine and Animal Production, Naples, Italy;; b Pathology Unit of the Department of Veterinary Medicine and Animal Production, Naples, Italy;; c Interdepartmental Radiology Centre, University of Naples “Federico II”, Naples, Italy

**Keywords:** Cats, feline, melanoma, ocular neoplasia, phthisis bulbi

A 13-year-old, spayed, female Domestic Shorthair cat was presented for evaluation of buphthalmia (enlargement of the eyeball with an increase in the intraocular pressure) of the right eye, which according to the owner, had developed over the previous month. The cat had been adopted when it was a stray, 3-month-old kitten. At the time of adoption, the right eye was blind, microphthalmic, hypotonic, and showed advanced diffuse opacity; a diagnosis of *phthisis bulbi* had been speculated.

Examination of the right eye showed buphthalmia, moderate clear discharge, and a large pink/greyish neoplastic mass involving the entire ocular surface. The neoplastic tissue precluded visualization of the intraocular structures and the measurement of intraocular pressure (IOP). Examination of the left eye was unremarkable, and the complete physical examination was normal. The differential diagnoses for the right eye lesion included feline ocular sarcoma, melanoma, adenocarcinoma, lymphosarcoma, and squamous cell carcinoma. However, benign ocular growth, like a leproma (Lamagna et al. 2009) had to be taken into consideration.

**Figure 1.**
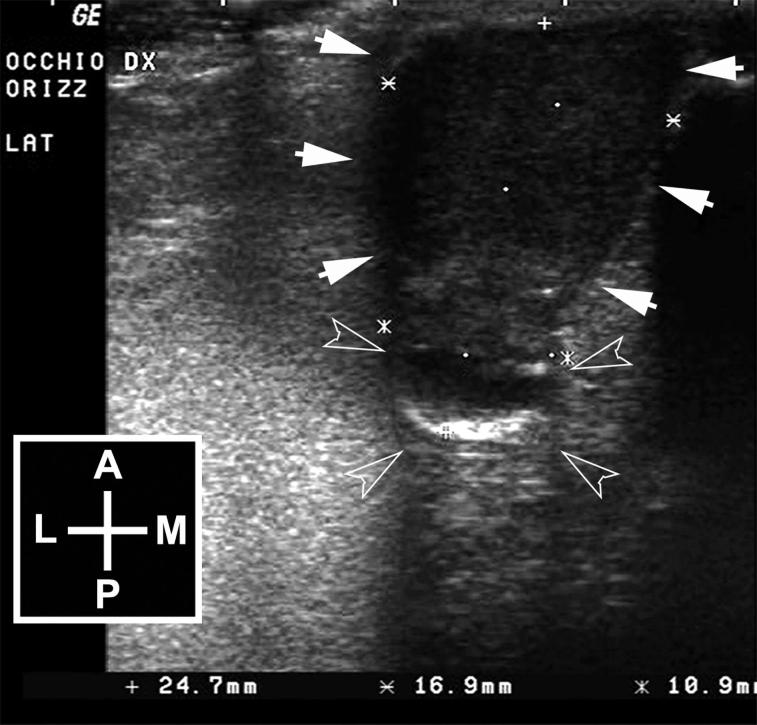
Horizontal ultrasonographic scan of the right eye. The eye shows a “hourglass” shape with a larger echoic anterior lesion (arrows) that continues in a posterior rounded mixed-echoic portion, attributable to the vitreous chamber and the fundus (empty arrow heads). Legend: A = anterior; P = posterior; L = lateral; M = medial.

Ultrasonography was performed without sedation and the patient positioned in sternal recumbence, using a 13 MHz linear transducer (Logiq 400MD, General Electric, Milano, Italy) directly on the neoplastic globe, following the application of topical anesthetic and sterile ultrasound coupling gel (Sterile Aquasonic 100©; Parker Lab. Inc., Fairfield, NJ, USA). Ultrasonography demonstrated a severe distortion of the right eye that had an “hourglass” shape with an anterior, larger, echoic, and highly vascularized oval lesion, and a posterior, smaller, mixed echoic, rounded portion. The posterior portion showed a peripheral hyperechoic line, compatible with the retinal fundus, and an anechoic content, compatible with the vitreal body, partially invaded by the anterior lesion (Figure 1).

The ocular signs and ultrasonographic findings were consistent with an intraocular neoplasia of a phthisical eye.

A complete blood cell count, serum biochemistry profile, thoracic radiographs, and abdominal ultrasonography were performed and showed no abnormal findings. Feline Leukemia Virus (FeLV) and Feline Immunodeficiency Virus (FIV) indirect immunofluorescence assays were negative.

Analgesia was provided with 0.01 mg/kg BW buprenorphine SQ, and a fine-needle aspiration (FNA) of the affected eye was performed: cytologic examination revealed moderate blood contamination and the presence of numerous macrophages, eosinophils, mast cells, and rare lymphocytes and plasma cells. Further, there was a population of cells having a spindle-to-oval shape, variable diameter, moderately basophilic amelanotic cytoplasm, with poorly defined margins and tail extensions. These cells had round-to-oval nuclei with multiple prominent nucleoli. Cells containing dark granules were rarely observed. Retromandibular lymph node cytology, obtained by FNA, was unremarkable.

Exenteration of the affected orbit was performed via a transpalpebral approach and the ocular globe and the surrounding mass were submitted for histologic examination.

On gross examination, the enucleation specimen consisted of two crescent shaped proliferations of tissue with diameters measuring 1.5 cm and 1 cm, respectively.

**Figure 2.**
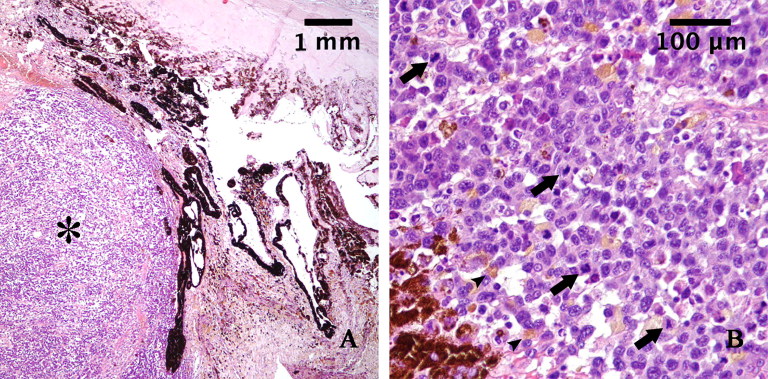
A: Histopathological section of the tumor mass stained with hematoxylin and eosin (magnification: 4×): Iris is infiltrated by a densely cellular neoplastic proliferation (asterisk). B: Histopathological section of the tumor mass stained with hematoxylin and eosin (magnification 40×): The neoplastic cells appear pleomorphic, rarely pigmented (arrowheads), with high nucleus-to-cytoplasm ratio and show numerous mitoses (arrows).

The right eye and the anterior neoplastic lesion were fixed in 10% buffered formalin and embedded in paraffin wax. Histologic examination was conducted on 5-μm-thick hematoxylin-eosin (HE)–stained sections. Histologically, the neoplasm consisted of an unencapsulated mass infiltrating the iris and the ciliary body (Figure 2(a)). The proliferation was composed of sheets, bundles, and nests of round and spindle cells, of variable dimensions, associated with a modest fibrovascular stroma. The cells showed moderate amounts of eosinophilic cytoplasm with poorly defined borders, sometimes vacuolated; less than 10% of the cells contained brown pigment granules (melanin). The cells had eccentric round or oval nuclei with clumped chromatin and one or two prominent nucleoli. Karyomegalic and cytomegalic forms were common, and nuclear pseudo-inclusions were observed. There was severe anisokaryosis and anisocytosis. Atypical mitotic figures were frequent, mitotic index was 7 mitoses in ten 40x-high-power fields (HPF) (Figure 2(b)).

**Figure 3.**
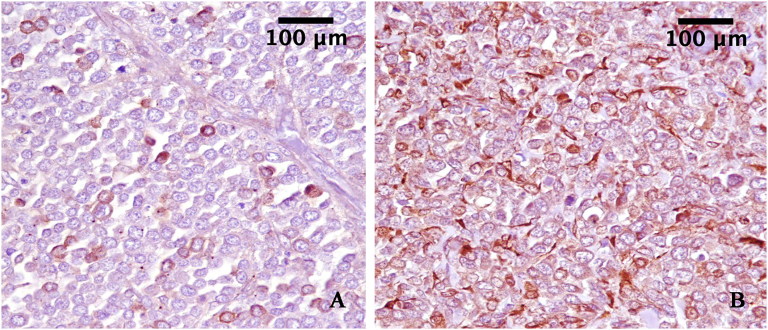
A: Neoplastic cells expressing Melan-A (3,3′-Diaminobenzidine indirect immunohistochemistry. Hematoxylin in counterstain) with strong label intensity in some cells. (original magnification 40×). B: Neoplastic cells expressing S100 (3,3′-Diaminobenzidine indirect immunohistochemistry. Hematoxylin in counterstain) with strong label intensity diffuse in cellular proliferation. (original magnification 40×).

Immunohistochemical staining was performed on sections using a commercial Kit MACH1, (Biocare Medical LLC, Concord, California, USA). Briefly, the sections were incubated overnight in a humidified chamber at 4 °C with antibodies anti-S100 and anti-Melan A (Dako, California, USA), diluted to 1:200. To reveal the immunolabelling, diaminobenzidine (DAB) was used as a chromogen and the sections were counterstained with hematoxylin. The immunohistochemical staining with anti-Melan A antibody showed a strong immunoreactivity in several neoplastic cells. A strong immunolabelling for S100 antibody was observed in all neoplastic cells. (Figure 3).

The diagnosis of diffuse iris melanoma was made based on these observations. The cat was discharged with 15 mg/kg BW of amoxicillin/clavulanic acid PO q12 h for 7 days and meloxicam 0.1 mg/kg BW PO q24 h for 3 days. The patient had an uneventful recovery, and the exenteration wound healed without complication within 10 days.

**Figure 4.**
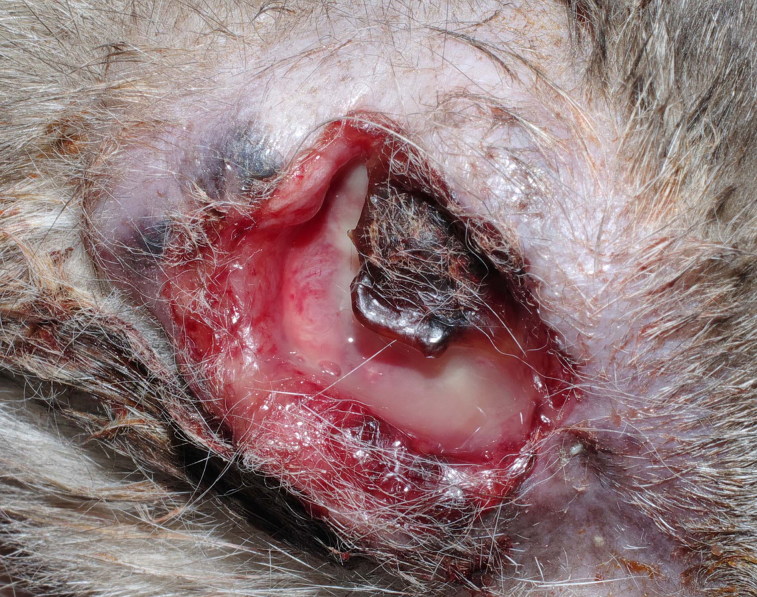
The cat, 14** **months after surgery. Neoplastic tissue invading orbital space and developing over the site of the exenteration scar, associated with cutaneous necrosis and ulceration, can be observed.

The owner elected not to pursue further anti-cancer therapy; 6 months postoperatively, the cat was clinically healthy with no sign of recurrence or metastasis. Seven months after surgery, the referring veterinarian reported that a small lesion over the scar on the site of exenteration had developed but owners declined any diagnostical procedure and further treatment. Two months later, the mass had enlarged with cutaneous necrosis and ulceration (Figure 4). Fourteen months postoperatively, the cat died.

Necropsy revealed enlarged regional lymph nodes and some nodular lesions in the lungs, sized 0.1–3 mm. Samples were collected from various lymph nodes, brain, lungs, heart, stomach, liver, pancreas, intestine, kidneys, and urinary bladder. Histological examination of the samples confirmed the presence of metastases in the regional lymph nodes and lungs. Furthermore, micro-metastases were detected in regional lymph nodes, lung, liver, and kidneys.

To the best of our knowledge, this report describes the first case of melanoma arising in phthisis bulbi in a cat. Phthisis bulbi denotes an end-stage eye disease characterized by shrinkage and disorganization of the eye with resultant functional loss (Tripathy et al. 2018). Eyes presenting with phthisis bulbi are blind, small, opaque and painless, therefore they are frequently not monitored. However, clinicians and pathologists should be aware of rare malignancies that may present in phthisical eyes. Therefore, monitoring and early enucleation of eyes of cats with phthisis bulbi should be considered.

Tumors of melanocytic origin are the most common primary intraocular neoplasms reported in cats (Williams et al. 1981; Dubielzig et al. 1990; Stiles 2013; Finn et al. 2008). Iris melanoma is the most frequent feline intraocular tumor, with the classical presentation of a progressive diffusion, in the iris, of a focal area of pigmentation that may affect other ocular structures (ciliary body, choroid, iridocorneal angle, sclera) and lead to uveitis and glaucoma. However, rare cases of choroidal melanocytoma (Semin et al. 2011), or choroidal melanoma (Harris and Dubielzig 1999; Bourguet et al. 2015) have been reported.

In cats, enucleation is the treatment of choice for malignant melanocytic tumors and for blind or painful eyes (Dubielzig 1990; Finn et al. 2008). Although metastatic rates of 24-63% have been reported (Patnaik and Mooney 1988; Duncan and Peiffer 1991), adjunctive therapy protocols following enucleation have not been established yet. The complications after enucleation in cats are not usually serious, though orbital or conjunctival tissue swelling may develop infrequently weeks to months postoperatively, due to retained lacrimal and/or nictitating membrane glandular tissues (Gelatt and Gelatt 2001; Trio et al. 2004; Spiess 2007). Orbital emphysema is a rare complication caused by retrograde movement of air through a patent nasolacrimal system (Meomartino et al. 2015).

In the current case, the unusual finding of a non-pigmented ocular melanoma, associated with *phthisis bulbi*, focuses attention on the histological characteristics and prognostic evaluation.

The neoplastic cells had marked atypia, necrosis, and a mitotic index of 7 per 10 HPF which were indicative of malignancy based on the literature (Duncan and Peiffer 1991; Kalishman et al. 1998; Wiggans et al. 2016); further, neoplastic invasion of the ciliary body and blood vessels, and extrascleral extension have been associated with decreased survival in cats affected with diffuse iris melanoma (Duncan and Peiffer 1991; Kalishman et al. 1998; Wiggans et al. 2016).

Recently, some immunohistochemical parameters helpful in determining cats at risk of metastasis of diffuse iris melanoma were reported, and increased melan-A label intensity was associated with increased rate of metastasis (Wiggans et al. 2016).

In our case, the malignant histological characteristics of the tumor and the results of immunohistochemistry, with a strong immunoreactivity in several neoplastic cells for anti-Melan-A and S-100 antibodies, suggested a poor prognosis. The owner declined adjunctive therapy, however, future studies should examine the efficacy of adjunctive chemotherapy or radiotherapy in treatment of ocular tumors presenting with these characteristics of malignancy in felines.

Only three cases of ocular tumor associated with phthisis bulbi in cats have been identified in the literature (Perlmann et al. 2011; Woog et al. 1983). In 2011, Perlmann et al. reported two cases of feline intraocular sarcomas in cats with phthisis bulbi (Perlmann et al. 2011): in these cases, the diagnosis was based on the immunohistochemical results (negative for CD3, CD79a, cytokeratin, and S-100, and positive for vimentin). It had been suggested that chronic intraocular inflammation preceded the development of intraocular sarcoma in these cats (Perlmann et al. 2011). Feline intraocular sarcoma, in fact, affects cats one to ten years from an initial trauma, but also cats with chronic uveitis with no history of trauma (Dubielzig et al. 1990; Dubielzig 2008).

One report of osteosarcoma occurring in association with phthisis bulbi in a cat has been described (Woog et al. 1983). It was hypothesized that the affected cat had intraocular bone formation, often associated with *phthisis bulbi*, and that the heterotopic bone underwent sarcomatous change (Woog et al. 1983).

In these above-mentioned cases of feline ocular neoplasm associated with phthisis bulbi a significantly reduced life expectancy for affected cats was reported, but, unfortunately, the lack of diagnostic imaging at follow-up or necropsy did not permit an adequate assessment of tumor metastatic behavior (Perlmann et al. 2011; Woog et al. 1983). However, neurologic symptoms occurred briefly after surgery and a central nervous system invasion by neoplastic cells from the eye was suspected (Perlmann et al. 2011; Woog et al. 1983).

In humans, it has been long known that a phthisical eye may harbor an occult malignant melanoma, though very few such cases, occurring in the choroid, have been reported (Sarma et al. 1983; Perry et al. 1977; Tripathi et al. 2002). Sarma et al. 1983 reported a case of malignant melanoma in the left eye of a 62-year-old man who had been blind due to trauma for 35 years. Perry et al. (1977) reported a case of occult choroidal malignant melanoma in an eye with spontaneous expulsive choroidal hemorrhage. Tripathi et al. (2002) reported a case of choroidal melanoma associated with massive retinal gliosis in a painful and phthisical eye of a 45-year-old woman who had undergone bilateral surgery for congenital cataracts at the age of about 3 months and developed phthisis in the right eye and secondary glaucoma in the left eye.

In our case, the preliminary diagnosis of intraocular neoplasm was made on the basis of ophthalmic examination and ultrasonography. Thereafter, FNA of the affected eye was performed. It has been demonstrated that the accuracy of ultrasonography in diagnosing intraocular neoplasia is high in veterinary medicine (Gallhoefer et al. 2013). In humans, A-scan echographic characteristics of choroidal and ciliary body malignant melanomas permit the prediction of histopathological findings in > 95% of cases and, similarly, it has been demonstrated in spontaneous anterior uveal melanomas in dogs that A-mode evaluation may help to differentiate melanomas from other tumor types (Baptista et al. 2006). Further studies are needed to evaluate diagnostic and prognostic indicators, by ultrasound biomicroscopy and calibrated power spectrum, in the examination of feline intraocular tumors.

This case report highlights that development of intraocular melanoma in association with phthisis bulbi represents a rare finding in cats. Therefore, this tumor has to be considered when a blind and hypotonic eye suddenly becomes buphthalmic. In our case, ultrasonography allowed a tentative diagnosis of an intraocular tumor with high accuracy, without the need for anesthesia. Immunohistochemistry confirmed morphological and histological diagnosis and showed that it may be highly useful for prognostic purposes in clinical practice.
